# Antecedents

**Published:** 1857-04

**Authors:** 


### ANTECEDENTS.

SELDOM has a more important lesson been taught to parents, than in the last Presidential election: lessons loud enough to ring through creation—almost loud enough to wake the slumbering dead to life.

Father! teach your son, that he may be just stepping into the first office of the nation, and fail! because of some remembered meanness of school-boy life; or some ground for charge that "*honor bright*" was wanting in some early transaction. Tell him never to do an act which, although admissible in itself, may bear the impress of fraud without distressingly nice distinctions.

Mother! teach your daughters that the acts of their youth may be brought before the scrutiny of the millions of a generation yet unborn; and that never, for any cause, should they allow themselves to be placed in a questionable position; to labor to stand every hour of their existence right under the blaze of a noon-day sun.

All, both men and women, boys and girls, have a "*right*" to do multitudes of things which, at the same time, are not expedient. The greatest apostle of Christianity had a right to eat meat, yet he avoided it, for policy's sake. He resigned his own rights heroically, that he might make others happier thereby. That is the truest philanthropy. The person who will have his rights, suffer who may, has the first, chiefest want of a manly heart. Such an one can never attain immortal renown—can never rise above the vulgar herd.

One candidate for a high legislative honor, found the unjust act of many years agone, when he was friendless and obscure, thrown right across the door, and a more favored one entered in. A man of senatorial dignity was charged with a theft committed at school, in the days of his childhood. Another, of no mean repute, of many kindly memories, was found, shall we say "almost ages ago," helping himself in his neighbor's wine cellar. In all these cases there may have been circumstances to palliate, to warrant, to justify, if they were critically examined; but the masses despise niceties; they love to see a man's conduct stand out *alto relievo.* They want to see him right at a glance.

Do not forget that it is not always enough to act in such a way as to be right. Some actions are proper enough in themselves, but are liable to misinterpretation. A man may be seen coming out of a common grog-shop: he may have gone in there to get a glass of water, but it was an act largely capable of a vicious interpretation. Teach your children as far as possible, that their actions should be so clearly proper as to need no interpreter; that they should never do a deed, or speak a word or write a line, which they would rather not have their parents know. One of the first steps from childhood to ruin is concealment from parents. One of the most valuable points to be gained by parents, is to secure the confidence of their children. No daughter was ever lost who confided wholly in her mother's heart. No young man ever went to ruin, who made his father his most intimate friend.

---

WHAT is worth doing at all, is worth doing well.

